# Exploring associations of perceived mental fatigue and perceived energy with gait characteristics during level and downhill walking in healthy young adults

**DOI:** 10.3389/fspor.2026.1973398

**Published:** 2026-10-23

**Authors:** Thomas Jürgen Klotzbier, Nadja Schott

**Affiliations:** Department of Sport and Exercise Science, University of Stuttgart, Stuttgart, Germany

**Keywords:** elevation at midswing, gait control, locomotion, mental fatigue, spatio-temporal gait parameters, trunk range-of-motion, working memory task, young adults

## Abstract

Mental fatigue may influence locomotor control, but evidence in healthy young adults is limited. This study examined whether a 40-minute working-memory task was accompanied by changes in perceived fatigue and cognitive performance and whether subjective fatigue and energy states were associated with pre-to-post changes in gait during level and downhill treadmill walking. Forty healthy young adults were included in the analyses and randomly assigned to a low-load 0-back condition (*n* = 21) or a high-load 2-back condition (*n* = 19); questionnaire-based analyses used the 38 participants with complete data. We assessed mental and physical energy and fatigue immediately before and after the task using the Mental and Physical State and Trait Energy and Fatigue Scales. We recorded reaction time and accuracy across five task blocks. Gait was assessed with six inertial sensors before and after the task during level walking (0°) and downhill walking (9° decline) at 5 km/h. Associations between subjective fatigue and energy ratings and pre-to-post changes in gait parameters were examined using Pearson correlations. Perceived mental fatigue increased and perceived mental energy decreased after the cognitive task. Perceived physical fatigue also increased, whereas perceived physical energy did not change significantly. Reaction times improved across blocks in both conditions; accuracy decreased in the 0-back condition and increased in the 2-back condition. The divergent subjective and behavioral responses indicate increased perceived mental fatigue without convincing evidence of cognitive performance fatigability. Most exploratory fatigue-gait correlations did not reach nominal significance. During downhill walking, perceived mental fatigue showed a small-to-moderate exploratory association with elevation at midswing (*r* = 0.329, unadjusted *p* = 0.044). Exploratory associations were also observed between trunk coronal range of motion and subjective physical energy, physical fatigue, and mental energy during downhill walking, and between trunk coronal range of motion and mental energy during level walking. These correlations indicate covariation between subjective states and lateral trunk movement but do not provide evidence of impaired stability. No association remained significant after false-discovery-rate correction across all 200 correlations. In healthy young adults, perceived fatigue and energy states showed selective exploratory associations with changes in trunk and foot movement rather than widespread associations with gait parameters. The associations involving trunk coronal range of motion may reflect an altered movement strategy or normal movement variability; without direct measures of dynamic stability, their functional relevance cannot be determined. Similarly, the association with elevation at midswing raises the hypothesis that perceived mental fatigue may relate to a more cautious foot-trajectory strategy. This hypothesis could not be tested with the present measurements and requires confirmation with direct biomechanical and stability measures; the finding does not establish a safer or compensatory gait adaptation. Larger, preregistered studies with correction for multiple comparisons and direct biomechanical and stability measures are needed.

## 1 Introduction

Fatigue-related impairments may contribute to accidents and falls, particularly in occupational settings. Physical exertion can impair motor performance and movement control (1, 2). Helbostad and colleagues (2) investigated the effects of physical fatigue on balance control during walking in older adults. Following a repeated sit-to-stand task, participants showed significant increases in step width (*p* = 0.023) and mediolateral trunk acceleration amplitude (*p* = 0.038). The authors therefore suggested that physical fatigue may represent a risk factor for falls in older adults. More recent findings indicate that fatigue-related changes in gait and balance may also be relevant in younger populations and occupational contexts. Allin and Madigan (3) examined 36 healthy young adults after two hours of simulated manual material handling. Although the heavy and light workload conditions did not differ significantly in trip- or slip-related fall risk, the combined analysis indicated less stable gait, an increased risk of slipping while carrying a load, and impaired balance recovery following a laboratory-induced trip. Furthermore, Zhang and colleagues (4) found that experimentally induced muscle fatigue altered lower-limb kinematics and muscle activation during walking in healthy adults. Fatigue was associated with reduced ranges of motion, including smaller plantarflexion, knee flexion, and hip flexion angles, as well as increased muscular activation. These changes may reflect compensatory adaptations that maintain stability despite fatigue-related impairments in movement control.

Physical fatigue may also affect cognitive performance. Eddy et al. (5) showed that subjective physical fatigue increased during prolonged physical exertion, whereas cognitive performance decreased. Participants walked on a treadmill for two hours while carrying either no additional load or a 40-kg load. Cognitive performance on an auditory go/no-go task deteriorated over time in the loaded condition relative to the unloaded condition, as indicated by an increased proportion of false alarms. Collectively, these findings suggest that prolonged physical exertion may simultaneously affect cognitive functioning, motor control, and gait stability.

Prolonged periods of demanding cognitive activity, such as monitoring production processes, controlling machinery, or performing repetitive administrative tasks, can induce mental fatigue. Mental fatigue is commonly described as a psychobiological state characterized by subjective feelings of tiredness, reduced energy, and an increased effort required to maintain task performance (6, 7). Mental fatigue has been associated with reduced attention, increased distractibility, impaired performance monitoring, and less efficient response selection (8). However, its effects are not restricted to cognitive performance and may extend to motor and physical performance. In their systematic review and meta-analysis, Brown et al. (9) reported a significant small-to-medium negative effect of prior cognitive exertion on subsequent physical performance. Cognitive tasks lasting less than 30 min (*g* = −0.45) and those lasting 30 min or longer (*g* = −0.30) produced comparable negative effects. Similarly, Van Cutsem and colleagues (10) concluded that mental fatigue primarily impairs endurance performance, whereas maximal strength, power, and anaerobic performance appear to be less consistently affected. The magnitude of impairment may depend on the duration and intensity of both the preceding cognitive task and the subsequent physical task. An increased perception of effort has been proposed as a principal mechanism underlying the negative effect of mental fatigue on endurance performance (10). More recent meta-analytic evidence has generally confirmed a detrimental effect of mental fatigue on endurance performance, although substantial heterogeneity between studies remains and no single methodological or individual factor appears to explain the variability in outcomes (11).

Researchers have examined the potential effects of mental fatigue on gait and postural control mainly in older adults or clinical populations. Evidence in healthy young adults remains comparatively limited and inconsistent. Mental fatigue temporarily changes the availability or allocation of cognitive resources and may therefore affect gait and postural control when the motor task requires substantial attentional involvement (12). Locomotion is no longer considered an exclusively automatic motor activity but rather a task that also relies on executive functioning, attention, sensory integration, and anticipatory control (13). This cognitive involvement increases when individuals encounter complex or changing environmental conditions, perform an additional task, or walk on demanding surfaces.

Because mental fatigue may reduce the allocation of attentional resources to a subsequent task (14), it could compromise the ability to maintain balance during complex walking conditions. However, recent findings in young adults indicate that the effects depend strongly on the difficulty of the balance or locomotor task. A systematic review by Brahms and colleagues (15) found an overall negative effect of mental fatigue on balance performance, with more pronounced effects during demanding balance conditions. In contrast, individual experimental studies have produced mixed findings. Gebel et al. (16), for example, observed increased variability in center-of-pressure measures following mental fatigue in healthy young adults, but the effects on mean postural sway were smaller than those induced by physical fatigue. Similarly, Weissinger and colleagues (17) found only marginal effects of perceived cognitive fatigue on static postural control, suggesting that relatively simple balance tasks may remain largely automatic in healthy young adults. These findings indicate that sufficiently challenging motor tasks may be required to reveal fatigue-related impairments in this population.

Inclined walking may provide such a challenging condition because it requires continuous adaptation of lower-limb kinematics, postural orientation, and braking or propulsive forces. Downhill walking is particularly demanding because it involves eccentric muscle activity and increased requirements for controlling forward progression and maintaining dynamic stability. Consequently, fatigue-related reductions in cognitive control may appear as changes in spatio-temporal gait parameters or compensatory strategies, such as adjustments in stride length, cadence, step width, or double-support time. Despite its practical relevance, little is known about how experimentally induced mental fatigue affects gait during uphill and downhill walking in healthy young adults. Investigating this relationship may improve our understanding of whether mental fatigue contributes to movement adaptations that could become relevant to accident and fall risk under demanding occupational or everyday walking conditions.

Therefore, the aims of the present study were: (1) to determine whether a 40-min working-memory task was accompanied by changes in subjective fatigue and energy as well as cognitive task performance, and (2) to examine, in a correlational and exploratory analysis, whether individual differences in subjective fatigue and energy were associated with pre-to-post changes in spatio-temporal gait parameters during level and downhill treadmill walking.

We hypothesized that subjective mental fatigue would increase and mental energy would decrease following the working-memory task. We further hypothesized that greater subjective mental fatigue would be associated with shorter stride length and longer double-support time, with stronger associations expected during downhill than during level walking.

## 2 Materials and methods

### 2.1 Participants

Forty healthy young adults volunteered for this randomized between-group pre-post study. Using random numbers generated with the RAND function in Microsoft Excel, participants were assigned to either the low-load 0-back condition or the high-load 2-back condition. All participants were recruited through word of mouth and contacts within the university community. They were informed about the nature and aims of the study and provided written informed consent. All procedures were conducted in accordance with the Declaration of Helsinki and applicable ethical standards, legal requirements, and international norms (18). The University of Stuttgart internal ethics committee approved the study.

### 2.2 Measures

#### 2.2.1 Sociodemographic information, health characteristics, and physical activity

Sociodemographic and health-related characteristics included age, gender, body mass index (BMI), and physical activity. Physical activity was quantified in minutes per week. Participants were asked how many training sessions they currently completed per week and how long each session lasted. Based on this information, total weekly physical activity (minutes) was calculated.

#### 2.2.2 Subjective fatigue ratings

Subjective fatigue ratings were obtained using Part I of the Mental and Physical State and Trait Energy and Fatigue Scales (MPSTEFS) (19), based on the instruction “How do you feel right now?”. Part I comprises six items assessing feelings of energy and fatigue related to physical activity and six corresponding items assessing energy and fatigue related to mental activity. These 12 items were assigned to four subscales: physical energy, mental energy, physical fatigue, and mental fatigue, with three items per subscale.

Each subscale score was calculated by summing the ratings of its three items, each assessed using a 140-mm visual analogue scale (VAS), resulting in a possible total score ranging from 0 to 420 mm per subscale. For example, the physical-fatigue score combined participants' ratings of their current levels of “fatigue,” “exhaustion,” and “feeling worn out”. Higher scores indicated greater levels of the respective energy or fatigue dimension. Subjective mental and physical energy and fatigue ratings were assessed before and after the cognitive-load n-back task.

#### 2.2.3 Cognitive-load n-back task

To manipulate cognitive load and elicit different levels of perceived mental fatigue, an n-back task with two difficulty levels was programmed in E-Prime and modified according to Yun et al. (20). In the low-load condition (0-back), one of four letters (“a,” “b,” “c,” or “d”) was presented visually on a 17-inch monitor. Whenever the letter “a” appeared, participants were instructed to press the “L” key on the keyboard as quickly as possible. When one of the other letters (“b,” “c,” or “d”) appeared, participants were required to press the “S” key. They were instructed to respond as quickly and accurately as possible.

 In the high-load condition (2-back), one of the same four letters was presented in each trial. Participants were instructed to indicate whether the current stimulus matched the stimulus presented two trials earlier.

Each stimulus was presented for 500 ms, followed by an interstimulus interval of 1,500 ms during which a blank white screen was displayed (Figure 1). Reaction time and response accuracy were recorded within a 2,000 ms response window beginning at stimulus onset. Participants in both conditions first completed a practice block comprising 12 stimuli. Subsequently, they completed five blocks of 160 trials each. A one-minute break was provided between blocks. The total duration of the n-back task was approximately 40 min.

**Figure 1:**
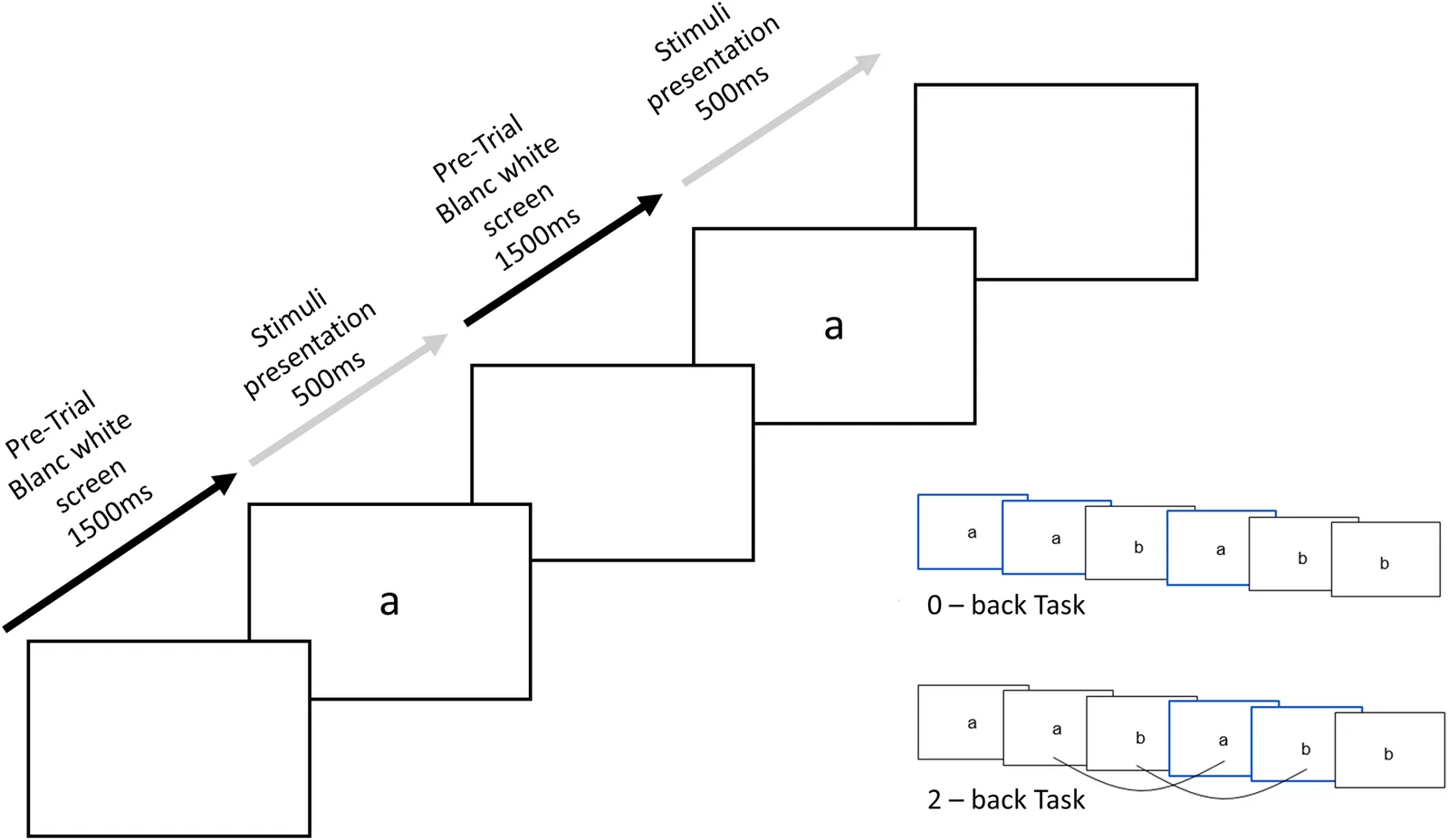
Timeline of the n-back task. The blue-highlighted stimuli in the lower-right area show the progression of the two difficulty levels.

#### 2.2.4 Treadmill walking protocol

Gait was assessed under two treadmill conditions: level walking at 0° and downhill walking at a 9° decline. The fixed speed of 5 km/h (1.39 m/s) was selected to standardize walking speed across participants, walking conditions, and pre- and post-task assessments. This speed is comparable to the 1.4 m/s used in previous inclined-treadmill research and approximates the preferred overground walking speed reported for healthy young adults (21). The 9° decline was selected to impose a meaningful biomechanical demand while remaining within the range of slopes used in previous experimental studies of hill walking. We did not individualize walking speed to participants' preferred walking speed.

For each walking condition, participants first completed a 40-s familiarization trial without data recording. Following a 10-s break, gait data were recorded during a second 40-s trial. Participants performed the same walking protocol during the pre- and post-task assessments. At both assessments, level walking always preceded downhill walking. Participants were instructed to look at a fixation cross positioned 2 m in front of the treadmill at a height of 1 m.

#### 2.2.5 Gait data acquisition and processing

Gait data were recorded at a sampling frequency of 128 Hz using six APDM Opal inertial measurement units and processed using Mobility Lab (APDM Inc., Portland, OR, USA; analysis version 2). The sensors recorded triaxial acceleration, angular velocity, and magnetic-field data.

The left-wrist and right-wrist sensors were attached to the dorsal aspect of each wrist between the ulnar and radial styloid processes. The left-foot and right-foot sensors were attached to the lateral aspect of each ankle, directly above the lateral malleolus. The lumbar sensor was attached to the lower back at the level of the L5 spinous process, corresponding to the midpoint of the line connecting the highest points of the iliac crests. The trunk sensor was attached over the sternum between the manubrium and the body of the sternum.

At both pre- and post-assessment, participants completed four walking trials, comprising two trials during level walking (0°) and two during downhill walking (−9°). Across all trials, walking conditions, and measurement time points, an average of 29.37 (*SD* = 3.68) steps per trial were included in the analysis. During the pre-assessment, an average of 29.71 (*SD* = 3.58) and 29.66 (*SD* = 3.34) steps were detected in the first and second level-walking trials, respectively. For downhill walking, the corresponding values were 29.73 (*SD* = 3.68) and 28.56 (*SD* = 4.81) steps. During the post-assessment, an average of 30.15 (*SD* = 2.98) and 29.34 (*SD* = 3.66) steps were detected during level walking, compared with 28.83 (*SD* = 4.64) and 29.00 (*SD* = 5.39) steps during downhill walking.

The proprietary Mobility Lab algorithms automatically detected initial contact and toe-off events using gyroscope signals from the bilateral lower-limb sensors. Initial contact represented the time at which the foot contacted the walking surface, whereas toe-off represented the time at which the foot left the walking surface. Temporal gait parameters were calculated from the detected gait events, and spatial gait parameters were estimated using the Mobility Lab biomechanical model. As Mobility Lab focuses on steady-state walking, steps occurring during acceleration and deceleration phases were excluded from the analysis. This approach enhances the comparability of the analyzed gait cycles and reduces potential biases associated with acceleration and deceleration effects.

Mobility Lab provided trial-level means based on the gait cycles automatically detected during each recorded 40-s trial. Only one recorded trial was analyzed for each combination of assessment time and walking condition; therefore, no averaging across repeated recorded trials was required. For parameters reported separately for the left and right sides, we averaged the two side-specific trial means to obtain a bilateral value for the statistical analyses. A complete overview of the operational definitions and units of all gait parameters is provided in Supplementary Material C.

### 2.3 Procedure

Testing was conducted in a quiet laboratory environment. Participants completed the entire assessment battery during a single experimental session. After providing written informed consent, we collected participants' sociodemographic and anthropometric data. Participants then completed the Mental and Physical State and Trait Energy and Fatigue Scales to assess baseline perceived physical and mental fatigue and energy. We then attached the inertial sensors to the predefined body locations.

Participants then completed the treadmill-walking protocol as the pre-task gait assessment. After the pre-task gait assessment, participants completed either the 0-back task in the low-load condition or the 2-back task in the high-load condition for approximately 40 min. Immediately after the n-back task, participants completed the fatigue and energy questionnaire again to assess their current physical and mental states. No scheduled rest or recovery period was provided. After completing the questionnaire, participants proceeded directly to the post-task gait assessment. The exact intervals between completing the n-back task and beginning the level- and downhill-walking assessments were not recorded. Participants then repeated the identical treadmill-walking protocol as the post-task gait assessment.

Walking speed, treadmill inclination, trial duration, familiarization period, sensor locations, and data-processing procedures were identical for the pre- and post-task assessments. At both assessments, level walking preceded downhill walking, resulting in the following fixed sequence: pre-task level walking, pre-task downhill walking, post-task level walking, and post-task downhill walking. Consequently, differences between walking conditions may reflect order effects in addition to treadmill inclination.

### 2.4 Data analysis

Statistical analyses were conducted using SPSS Statistics, version 27 (IBM Corp., Armonk, NY, USA). Initially, the dependent variables were screened for missing values, extreme values, and deviations from normality. Distributional assumptions were evaluated using Kolmogorov–Smirnov tests in combination with visual inspection of Q–Q plots and examination of skewness and kurtosis. For correlation analyses, we inspected scatterplots for linearity and potential outliers, and examined standardized residuals to identify potentially influential observations. Potential baseline differences between the experimental groups were examined using independent-samples *t*-tests for continuous variables and chi-square tests for categorical variables. The significance level was set at *α* = 0.05 for all statistical analyses (22). Cohen's *d* was calculated as the effect-size measure for independent-samples *t*-tests, and Cramér's *V* was calculated for chi-square tests.

Changes in physical and mental energy and fatigue following cognitive load were analyzed using a 2 × 2 × 2 repeated-measures analysis of variance (ANOVA), with domain (physical vs. mental), mood state (fatigue vs. energy), and time (pre vs. post) as within-subject factors (complete-case analysis, *n* = 38; two participants were excluded because of missing MPSTEFS data).

To examine changes in subjective fatigue and energy in response to the cognitive task, four separate 2 × 2 mixed-design analyses of variance (ANOVAs) were conducted for the MPSTEFS dimensions of mental fatigue, mental energy, physical fatigue, and physical energy. Time (pre-task vs. post-task) was included as a within-subject factor, while cognitive-load condition (0-back vs. 2-back) was included as a between-subject factor. The main effect of time was examined to determine whether subjective fatigue and energy changed from pre- to post-task across both conditions. The time×cognitive-load condition interaction was examined to determine whether the magnitude of these changes differed between the 0-back and 2-back conditions. To control the familywise error rate across the four dimension-specific interaction tests, *p*-values were adjusted using the Holm–Bonferroni procedure.

Reaction times and accuracy rates during the n-back task were examined using separate 5 × 2 repeated-measures ANOVAs, with block (blocks 1–5) as the within-subject factor and cognitive-load condition (0-back vs. 2-back) as the between-subject factor.

Pearson's bivariate correlation analyses were conducted to examine the relationships between post-assessment subjective fatigue ratings and changes in spatio-temporal gait parameters. Gait change scores were calculated as the post value minus the corresponding pre value. Change scores were selected because the primary aim of the correlation analyses was to quantify the direction and magnitude of within-participant gait changes and examine their associations with subjective fatigue and energy states. Correlations were calculated across all participants with available data, pooled across both cognitive-load conditions (*n* = 38 unless otherwise indicated; see the note to Table 1), separately for each walking condition.

**Table 1:** Sample characteristics, including mean values (standard deviation).

Characteristic	Low cognitive load (0-back)	High cognitive load (2-back)	Stat. analyses
	(*n* = 21)	(*n* = 19)	*p*-value; effect size
Age (years)	22.6 (2.01)	22.2 (2.29)	*0.559; 0.187*
Sex	11 M, 10 F	12 M, 7 F	.538; 0.109
BMI (kg/m^2^)	22.1 (2.45)	22.5 (2.11)	.559; −0.189
Physical activity (min/wk)	226 (95.1)	295 (199)	.170; −0.448

Sex distribution was assessed by a Chi^2^ test. For the Student t-test, effect size is given by Cohen′s *d*. For the Chi^2^ test, effect size is given by Cramer′s V.

No *a priori* sample-size calculation was conducted. A Fisher-z-based sensitivity analysis indicated that, with 38 complete cases, a two-sided *α* of 0.05, and 80% power, the minimum detectable correlation was approximately |*r*| = 0.44. In practical terms, the study had an 80% probability of detecting correlations of approximately *r* = 0.44 or larger in either direction. Smaller associations were less likely to be detected reliably. This value should not be interpreted as a significance threshold but as an indication of the effect sizes for which the available sample provided adequate statistical power.

No primary correlational outcome was prespecified; all correlation analyses were therefore exploratory, and *p*-values are reported unadjusted. To show how multiplicity affects the results, we additionally applied Benjamini-Hochberg false-discovery-rate (FDR) and Holm corrections to the *p*-values of all 200 correlations in Table 1 and report the adjusted values descriptively. Ninety-five percent confidence intervals for the nominally significant correlations were computed using the Fisher z transformation.

## 3 Results

### 3.1 Characteristics of the study population

A total of 40 healthy young participants (age 22.4 ± 2.17 years, 17 women) were randomly assigned to two cognitive-load conditions: low-load, 0-back (*n* = 21, *M* = 22.6, *SD* = 2.01, 10 female) and high-load, 2-back (*n* = 19, *M* = 22.2, *SD* = 2.29, 7 female) (see Table 2). The conditions did not differ significantly in the baseline characteristics. However, non-significant baseline tests in a small sample do not demonstrate equivalence, and the descriptive difference in physical activity (226 vs. 295 min/week; *d* = −0.45) should be noted.

**Table 2:** Correlations between post subjective fatigue and energy ratings and changes in spatio-temporal gait parameters, separated by walking condition.

Spatio-temporal gait parameter	Physical energy		Physical fatigue		Mental energy		Mental fatigue	
	Pearsons' *r*	*p*	Pearsons' *r*	*p*	Pearsons' *r*	*p*	Pearsons' *r*	*p*
Treadmill inclination 0° level								
Delta duration	−0.028	0.866	0.056	0.740	−0.166	0.319	0.242	0.144
Delta cadence	−0.025	0.884	0.137	0.411	0.085	0.613	−0.032	0.847
Delta double support	−0.030	0.860	0.101	0.546	−0.022	0.894	0.103	0.538
Delta elevation at midswing	0.016	0.922	−0.004	0.981	<0.001	0.995	−0.019	0.910
Delta gait cycle duration	0.004	0.980	−0.097	0.561	−0.082	0.623	0.052	0.754
Delta gait speed	−0.168	0.313	0.208	0.210	−0.032	0.849	0.117	0.486
Delta lateral step variability	−0.146	0.381	0.269	0.103	−0.049	0.772	0.081	0.628
Delta circumduction	−0.287	0.080	0.250	0.130	−0.195	0.240	0.249	0.131
Delta foot strike angle	−0.135	0.418	0.062	0.712	−0.114	0.496	0.142	0.395
Delta toe off angle	0.105	0.530	0.030	0.859	0.088	0.599	−0.106	0.527
Delta single limb support	0.047	0.780	−0.123	0.462	0.025	0.880	−0.113	0.501
Delta stance	−0.012	0.943	0.078	0.643	−0.019	0.908	0.092	0.582
Delta step duration	0.065	0.698	−0.152	0.363	−0.087	0.606	0.058	0.731
Delta stride length	−0.055	0.744	0.020	0.907	−0.068	0.683	0.090	0.591
Delta swing	0.012	0.943	−0.077	0.644	0.020	0.907	−0.092	0.582
Delta terminal double support	−0.003	0.984	0.079	0.638	0.008	0.962	0.082	0.623
Delta toe out angle	0.077	0.648	−0.070	0.678	0.124	0.459	−0.064	0.702
Delta lumbar coronal ROM	0.170	0.344	−0.209	0.243	0.109	0.545	−0.106	0.556
Delta lumbar sagittal ROM	−0.044	0.807	−0.012	0.947	0.085	0.637	−0.126	0.484
Delta lumbar transverse ROM	0.316	0.073	−0.300	0.090	0.225	0.208	−0.300	0.090
Delta trunk coronal ROM	−0.221	0.182	0.128	0.445	**−0**.345*	0.034	0.287	0.081
Delta trunk sagittal ROM	−0.180	0.278	0.014	0.933	−0.209	0.208	0.186	0.263
Delta trunk transverse ROM	−0.183	0.271	0.012	0.941	−0.280	0.088	0.315	0.054
Delta arm swing velocity	−0.126	0.452	0.192	0.248	−0.022	0.896	−0.038	0.821
Delta arm ROM	−0.109	0.513	0.112	0.502	0.040	0.813	−0.175	0.292
Treadmill inclination 9° downhill								
Delta duration	−0.218	0.189	0.109	0.516	−0.054	0.745	−0.017	0.921
Delta cadence	−0.057	0.732	0.048	0.777	−0.082	0.623	0.088	0.598
Delta double support	−0.067	0.690	0.065	0.698	0.162	0.331	−0.090	0.590
Delta elevation at midswing	−0.158	0.342	0.187	0.262	−0.252	0.127	**0**.329*	0.044
Delta gait cycle duration	0.036	0.829	0.009	0.956	0.107	0.521	−0.124	0.457
Delta gait speed	−0.214	0.197	0.216	0.193	−0.122	0.464	0.202	0.223
Delta lateral step variability	0.213	0.199	−0.155	0.354	0.047	0.781	−0.024	0.887
Delta circumduction	−0.291	0.076	0.218	0.188	−0.170	0.309	0.245	0.137
Delta foot strike angle	0.036	0.828	−0.158	0.344	0.041	0.805	−0.041	0.805
Delta toe off angle	0.174	0.297	−0.007	0.969	0.133	0.425	−0.074	0.661
Delta single limb support	0.083	0.619	−0.084	0.614	−0.152	0.362	0.082	0.623
Delta stance	−0.051	0.762	0.047	0.779	0.166	0.319	−0.094	0.575
Delta step duration	−0.022	0.897	−0.014	0.935	0.016	0.922	−0.048	0.773
Delta stride length	−0.077	0.647	0.086	0.609	−0.017	0.918	0.053	0.752
Delta swing	0.052	0.758	−0.047	0.778	−0.165	0.322	0.093	0.577
Delta terminal double support	−0.077	0.644	0.067	0.689	0.141	0.397	−0.074	0.660
Delta toe out angle	0.146	0.381	−0.112	0.502	0.142	0.396	−0.060	0.722
Delta lumbar coronal ROM	−0.220	0.219	0.161	0.371	−0.149	0.406	0.137	0.446
Delta lumbar sagittal ROM	0.114	0.529	−0.145	0.420	0.225	0.209	−0.192	0.285
Delta lumbar transverse ROM	0.076	0.674	−0.221	0.217	0.111	0.538	−0.214	0.231
Delta trunk coronal ROM	**−0**.452**	0.004	**0**.344*	0.035	**−0**.401*	0.013	0.319	0.051
Delta trunk sagittal ROM	−0.015	0.928	0.033	0.843	−0.023	0.892	−0.027	0.874
Delta trunk transverse ROM	0.129	0.442	−0.148	0.376	0.077	0.648	−0.111	0.507
Delta arm swing velocity	−0.225	0.174	0.163	0.329	−0.238	0.151	0.189	0.256
Delta arm ROM	−0.213	0.199	0.128	0.444	−0.172	0.301	0.095	0.572

*p < 0.05; **p < 0.01  ; Significant correlations are highlighted in **bold**; ROM, range of motion; delta, change between pre and post. The correlation analyses were exploratory. All reported *p*-values are unadjusted for multiple comparisons and should not be interpreted as confirmatory evidence. Therefore, emphasis should be placed on the effect-size estimates and their confidence intervals. Correlations were based on *n* = 38 participants unless otherwise indicated. For lumbar coronal, sagittal, and transverse ROM parameters, the available-case sample was *n* = 33 owing to missing data. Visual inspection of the Q–Q plots and examination of skewness, kurtosis, and standardized residuals indicated no severe distributional violations or outlying observations that substantially affected the reported associations.

### 3.2 Subjective fatigue and energy ratings pre- and post cognitive demand

Among the 38 participants with complete MPSTEFS data (two participants were excluded because they did not complete the questionnaire), we observed a significant interaction mood×time, F(1, 37) = 30.9, *p* < 0.001, *η*^2^p = 0.456, and a significant interaction domain x mood x time, *F*(1,37) = 11.9, *p* = 0.001, *η*^2^_p_ = 0.243, showing an increase in feelings of mental (*MD* = 74.4; *SE* = 11.7; *p* < 0.001) and physical fatigue (*MD* = 43.3; *SE* = 11.70; *p* = 0.01) and a decrease in mental (*MD* = −61; *SE* = 11.7; *p* < 0.001) and physical energy (*MD* = −22.2; *SE* = 11.7; *p* = ns; see Figure 2).

**Figure 2:**
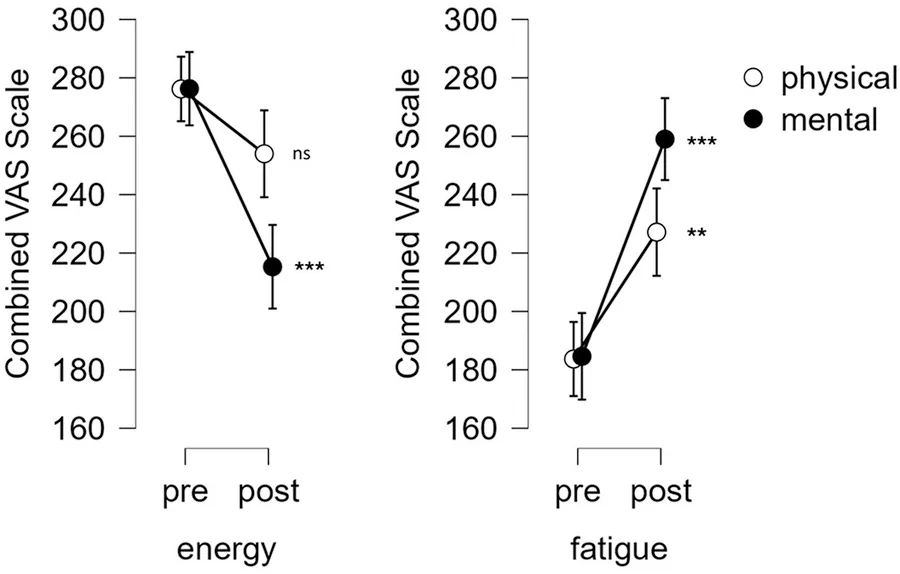
Change in perceived physical and mental energy and fatigue after cognitive engagement (**p < 0.01; ***p < 0.001; ns, not significant). Perceived energy and fatigue were assessed using 12 items distributed across four dimensions: physical energy, mental energy, physical fatigue, and mental fatigue. Each dimension comprised three items rated on 140-mm visual analogue scales (VAS). Item scores were summed to obtain a total score ranging from 0 to 420 mm for each dimension, with higher scores indicating greater perceived energy or fatigue, respectively.

Separate 2 × 2 mixed-design ANOVAs showed no significant main effects of cognitive-load condition on any of the subjective fatigue or energy dimensions (all *p* ≥ 0.065). Although pre-to-post changes were descriptively larger in the 2-back condition, the time×condition interaction was not significant for any dimension (mental fatigue: *p* = 0.068; mental energy: *p* = 0.108; physical fatigue: *p* = 0.190; physical energy: *p* = 0.671). These interactions also remained non-significant after Holm–Bonferroni correction (all adjusted *p* ≥ 0.272).

### 3.3 Performance in the working memory task throughout the 5 blocks

The results for correct response times showed a main effect group, *F*(1,38) = 104, *p* < 0.001, *η*^2^_p_ = 0.732, a main effect block, *F*(4,152) = 25.03, *p* < 0.001, *η*^2^_p_ = 0.397, and an interaction effect block x group, *F*(4,152) = 16.9, *p* < .001, *η*^2^_p_ = 0.308, indicating improved reaction times in both the 0-back and 2-back condition (see Figure 3a). The results for accuracy showed a main effect group, *F*(1,38) = 22.5, *p* < .001, *η*^2^_p_ = 0.372, a main effect block, *F*(4,152) = 2.98, *p* < .021, *η*^2^_p_ = 0.073, and an interaction effect block x group, *F*(4,152) = 9.00, *p* < .001, *η*^2^_p_ = 0.191, indicating decreased accuracy in the 0-back condition but improved accuracy in the 2-back condition (see Figure 3b). Because the 0-back and 2-back tasks differ in cognitive complexity and working-memory demands, the longer reaction times in the 2-back condition (main effect of condition) were expected, and absolute reaction times were not considered directly comparable between conditions. Cognitive performance was therefore evaluated mainly based on changes across task blocks.

**Figure 3:**
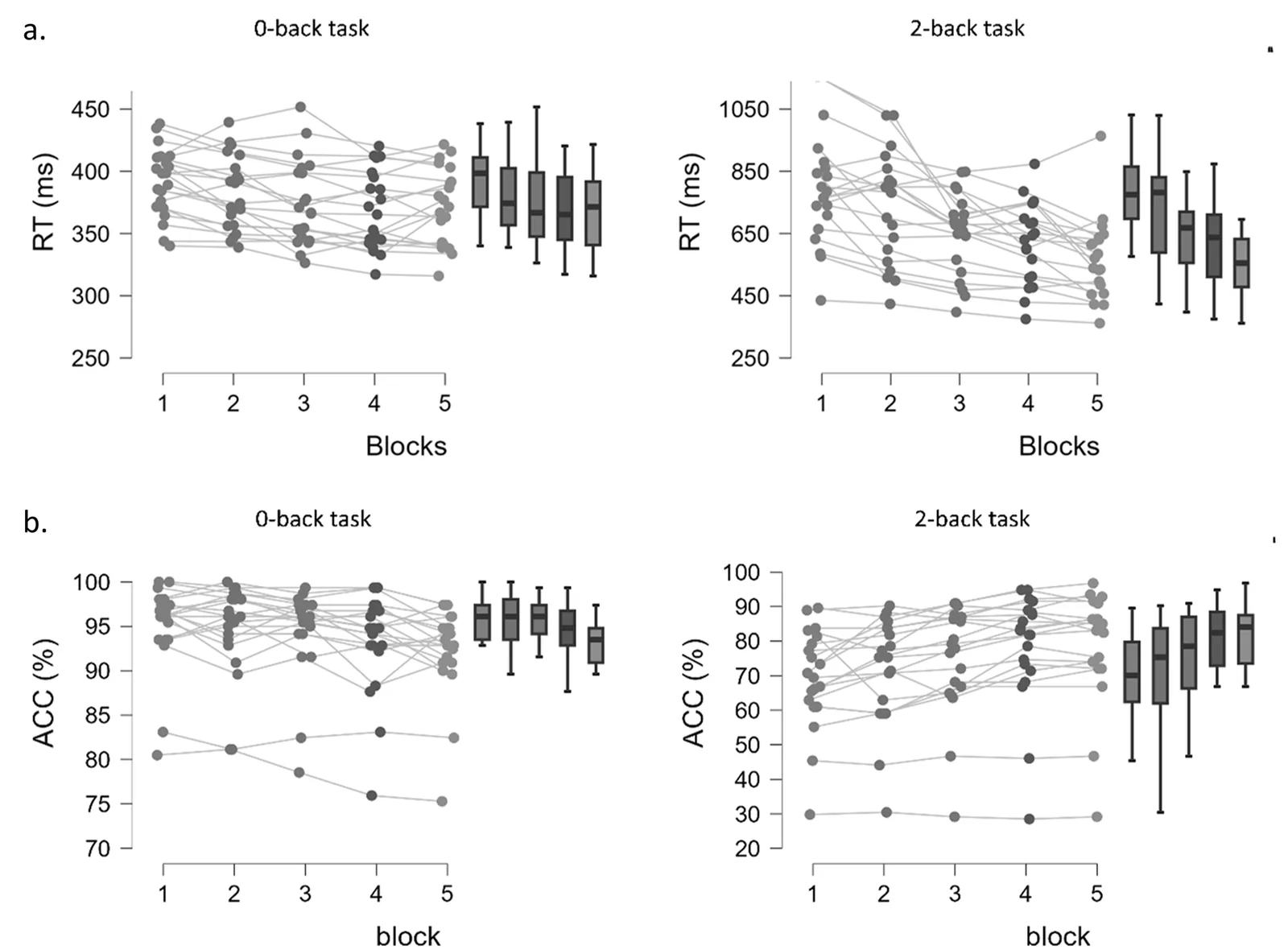
**(a)** Reaction times (ms) and **(b)** Accuracy (%) in the conditions of the n-back task across all 5 blocks.

### 3.4 Exploratory associations between post-assessment in subjective fatigue and changes in spatio-temporal gait parameters

Table 1 presents the exploratory associations between subjective fatigue and energy ratings and changes in spatio-temporal gait parameters during level (0°) and downhill (−9°) decline walking. The supplementary material (Supplementary Material A and B) contains the pairwise comparisons of correlations along with additional statistical information [effect size [Fisher's z] and confidence interval [95%]]. Among the exploratory analyses, perceived mental fatigue showed a nominal positive association with the change in elevation at midswing during downhill walking [*r* = 0.329, 95% CI (0.010, 0.587), unadjusted *p* = 0.044; see Figure 4]. Given the large number of comparisons, and because it did not survive correction for multiple testing, this association should be considered hypothesis-generating and may reflect chance. Of the 200 correlations, five (2.5%) had an unadjusted *p* < 0.05, which is fewer than the approximately ten expected by chance alone if all null hypotheses were true. The nominally significant correlations were: trunk coronal ROM with physical energy [downhill: *r* = −.452, 95% CI (−0.674, −0.155), *p* = 0.004], mental energy [downhill: *r* = −.401, 95% CI [−0.639, −0.093], *p* = 0.013; level: *r* = −.345, 95% CI [−0.599, −0.028], *p* = 0.034], and physical fatigue [downhill: *r* = 0.344, 95% CI (0.027, 0.598), *p* = 0.035]. After Benjamini–Hochberg FDR or Holm correction across all 200 tests, none of these associations remained significant (smallest adjusted *p* = 0.80); the same held when only the 100 downhill tests were considered (smallest FDR-adjusted *p* = 0.40). The confidence intervals are wide and several have lower bounds close to zero, indicating limited precision.

**Figure 4:**
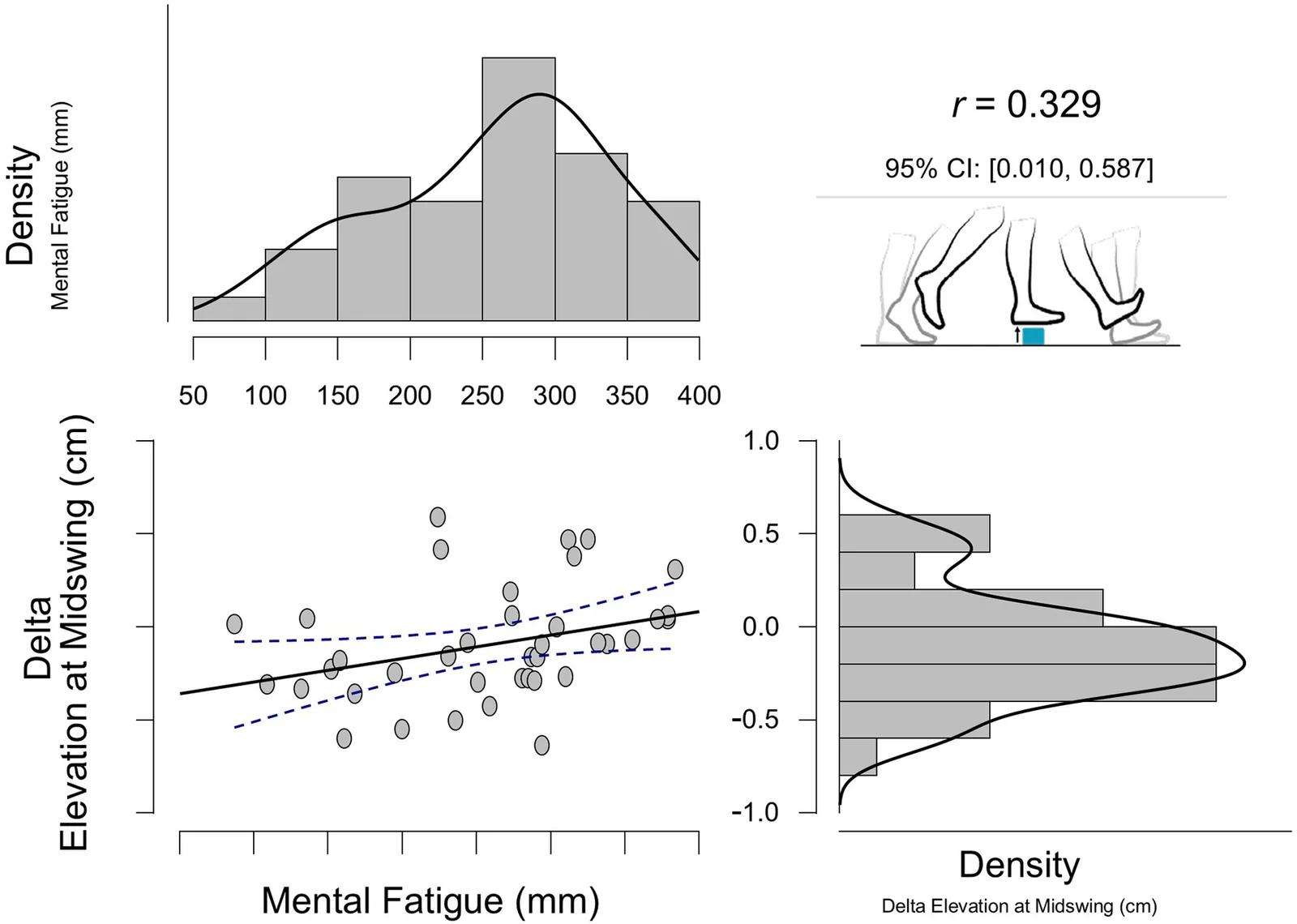
Scatter plot for the relationship between perceived mental fatigue and the change in elevation at midswing between pre and post (*n* = 38). The solid line is the fitted linear regression line and the dashed lines show its 95% confidence interval; *r* = 0.329, 95% CI [0.010, 0.587], unadjusted *p* = 0.044 (exploratory, not corrected for multiple comparisons).

## 4 Discussion

### 4.1 Perceived mental fatigue and cognitive performance

The present study examined whether a 40-min working-memory task was accompanied by increased subjective mental fatigue and whether subjective fatigue and energy states were associated with pre-to-post changes in gait during level and downhill treadmill walking. Participants reported greater perceived mental fatigue and lower mental energy after the n-back task, indicating that the task increased perceived mental fatigue. However, the objective performance data require a more cautious interpretation. Perceived fatigue and performance fatigability are related but distinct dimensions (23). Perceived cognitive fatigue refers to subjective tiredness and reduced energy, whereas cognitive performance fatigability requires an objective deterioration in performance over time. In the present study, reaction times improved across blocks in both n-back conditions. Accuracy decreased in the 0-back condition but improved in the 2-back condition. Thus, the findings do not demonstrate consistent cognitive performance fatigability. Practice, familiarization, or strategy adaptation may have counteracted the effects of perceived fatigue.

This dissociation is consistent with previous research showing that subjective fatigue can increase without a corresponding decline in cognitive performance (24, 25). The findings should therefore be described as evidence of perceived mental fatigue rather than unequivocal objective fatigability.

### 4.2 Associations between perceived mental fatigue and gait

Contrary to our hypothesis, perceived mental fatigue was not systematically associated with broad changes across the spatio-temporal gait parameters. Only a few nominally significant correlations emerged, and none remained significant after multiple-comparison correction. Because the sample was sensitive only to correlations of |*r|* ≥ 0.44, the absence of significant associations should not be taken as evidence that gait was maintained. This finding is consistent with the mixed literature. Kowalski et al. (26) similarly found that state fatigue and energy predicted selected gait characteristics in healthy young adults, although the explained variance was modest (3%–13%). Their findings support selective rather than widespread relationships between perceived fatigue and energy states and gait control.

Perceived mental fatigue has been more strongly associated with changes in gait and balance when tasks are cognitively demanding, particularly in older or clinical populations (12, 15). In healthy young adults, postural control may remain relatively robust. Gebel et al. (16) found increased variability in center-of-pressure measures after mental fatigue but no clear deterioration in mean postural sway, while Weissinger et al. (17) reported only marginal effects on static postural control. Recent gait studies similarly indicate that fatigue-related effects are selective rather than uniform. Piche et al. (27) found limited effects of mental and muscular fatigue on spatio-temporal dual-task gait parameters, except for selected measures of swing and single-support time. Schlegel et al. (28) also reported task- and age-specific treadmill-gait changes after sustained cognitive activity without a general decline in cognitive or gait performance.

Recent sports-biomechanics research extends this cognitive–motor relationship to more demanding lower-limb tasks. Wang et al. (29) reported that experimentally induced mental fatigue was accompanied by joint- and limb-specific changes in drop-landing biomechanics, while global lower-limb symmetry remained unchanged. Liu et al. (30) found that baseline cognitive performance moderated several ACL injury-related knee biomechanical responses during unanticipated sidestep cutting. Although drop landing and sidestep cutting differ substantially from treadmill walking, these findings further suggest that cognitive state and performance may be related to selective, task-dependent biomechanical responses rather than uniform deterioration across all movement parameters.

The constant treadmill speed of 5 km/h may have restricted adaptations in global parameters such as gait velocity. Fatigue-related responses may therefore have appeared through more subtle spatial adjustments. Although the nominal exploratory association between perceived mental fatigue and elevation at midswing was observed during downhill walking, this finding may reflect chance and does not establish that fatigue-related associations are stronger under downhill conditions.

### 4.3 Trunk coronal range of motion

Subjective energy and fatigue ratings were associated with trunk coronal ROM, which reflects lateral trunk movement in the frontal plane. Such movement helps control the body's center of mass relative to the base of support. However, increased trunk motion cannot automatically be interpreted as either impaired or improved stability.

Buurke et al. (31) found that reducing trunk motion in healthy adults decreased step width and mediolateral center-of-mass excursion without changing the mediolateral margin of stability. This demonstrates that trunk motion, step width, and center-of-mass control are functionally coupled. The association between higher fatigue, lower energy, and greater trunk coronal ROM in the present study may therefore reflect an altered movement strategy rather than a direct loss of stability. This interpretation is partly consistent with Chen and Chou (32), who found that mediolateral gait control deteriorated after physical fatigue in young adults. Nevertheless, their physical-fatigue protocol differs from the cognitive-fatigue paradigm used here, limiting direct comparisons. Bernstein's degrees-of-freedom framework (33) offers one possible explanation. The motor system can achieve a task goal through different combinations of joint and segment movements. Movement variability may therefore be functional when it enables performance to be maintained under changing constraints (34). Greater lateral trunk movement could, speculatively, represent an alternative way of achieving the same gait task, but the present data cannot test this.

This compensatory interpretation remains tentative. The correlational design cannot show that fatigue caused the change in trunk ROM, and the study obtained no direct measures of dynamic stability. The study also included no older comparison group. It can therefore only be concluded that subjective energy and fatigue states were associated with lateral trunk movement. Whether this represents compensation, reduced stability, or normal movement variability requires further investigation. Because perceived physical fatigue also increased following the cognitive task and was associated with changes in trunk coronal ROM, these findings may reflect a broader subjective post-task fatigue state rather than an effect specific to perceived mental fatigue.

### 4.4 Elevation at midswing

We observed a nominal exploratory association between perceived mental fatigue and elevation at midswing during downhill walking. This finding did not result from a prespecified primary analysis and was not adjusted for multiple comparisons. It should therefore not be interpreted as robust evidence of an association. Elevation at midswing describes the vertical elevation of the foot during the middle of the swing phase. Adequate foot elevation supports safe limb advancement and may reduce the likelihood of unintended contact with the walking surface. Although a higher foot trajectory may provide additional clearance from the walking surface (35), this parameter is not identical to minimum toe clearance, which represents the smallest distance between the toe and the ground during the swing phase. Therefore, the observed increase may reflect a more cautious gait strategy, but it cannot be concluded that it directly reduced tripping risk. A small-to-moderate exploratory association was observed between perceived mental fatigue and elevation at midswing during downhill walking [*r* = 0.329, 95% CI (0.010, 0.587), unadjusted *p* = 0.044]. Although this pattern may support a cautious foot-trajectory strategy, the wide confidence interval and exploratory nature of the analysis preclude conclusions about a reliable biomechanical adaptation. This condition requires continuous braking and regulation of body progression, potentially increasing the relevance of foot-trajectory adjustments (36). Lew and Qu (37) similarly showed that mental fatigue can impair slip initiation, detection, and reactive recovery, although slipping and foot elevation involve different mechanisms. Although a higher midswing elevation could be compatible with a compensatory strategy, the present exploratory association does not establish such a mechanism. A higher foot trajectory might also be accompanied by greater energetic demands or increased hip and knee flexion, neither of which was assessed in the present study. Because we did not measure joint kinematics, muscle activity, or metabolic costs, its functional benefit remains unclear. Moreover, the finding should not be described as a “high-steppage gait,” which is a pathological gait pattern associated with neurological or peripheral nerve disorders. The present result shows a continuous variation in a single parameter among healthy adults.

The most defensible conclusion is that greater perceived mental fatigue was associated with a higher foot trajectory during downhill walking. This is compatible with a cautious or compensatory strategy but requires confirmation using direct measures of minimum toe clearance, joint kinematics, muscle activity, and responses to actual trips or slips.

### 4.5 Methodological considerations and limitations

Several limitations should be considered. First, subjective fatigue increased without a consistent deterioration in cognitive performance. The study therefore provides stronger evidence for perceived mental fatigue than for cognitive performance fatigability. Second, the 0-back task was not necessarily fatigue-free. Performing a repetitive task for 40 min may induce fatigue, boredom, or reduced arousal. Accordingly, the 0-back condition should be interpreted as a lower-load cognitive comparison condition rather than as a fatigue-free control condition. In addition, the cognitive-load condition was not included in the analysis of fatigue and energy ratings or in the gait analyses. The study therefore cannot show that the 2-back task produced a greater increase in perceived mental fatigue, or a different gait response, than the 0-back task, and it calculated correlations across both conditions combined. Future studies should include an appropriate control condition and assess boredom, sleepiness, motivation, and perceived effort. Third, gait “change scores” were based on a single recorded 40-s trial per walking condition and time point. The use of simple change scores may be influenced by measurement error, regression to the mean, and the reliability and correlation of the pre- and post-task measurements. Because the reliability of the change scores was not independently quantified, the observed correlations should be interpreted as exploratory. Future adequately powered studies should apply baseline-adjusted models or linear mixed-effects models to examine changes in gait over time. Fourth, although the fixed treadmill speed ensured standardized conditions across participants and measurement occasions, it may have imposed different relative demands depending on preferred walking speed, anthropometry, fitness, and treadmill familiarity. These differences may have contributed to variability in gait change scores and their associations with subjective fatigue and energy. Future studies should assess preferred walking speed and compare standardized with individually adjusted treadmill protocols. The fixed order of the level and downhill conditions means that differences between walking conditions cannot be attributed solely to treadmill inclination. No recovery period was scheduled, and the exact intervals between the end of the n-back task and the two post-task walking conditions were not recorded. Downhill walking always occurred after level walking, allowing more time for possible recovery from perceived mental fatigue, and repeated exposure to the treadmill protocol may have produced familiarization or learning effects. Future studies should use individualized speeds and randomized or counterbalanced walking conditions. Fifth, numerous correlations were calculated across fatigue ratings, gait parameters, and walking conditions. With 200 correlations, approximately ten nominally significant results would be expected by chance alone; five were observed, and none remained significant after FDR or Holm correction. The sample was also sensitive only to correlations of |*r*| ≥ 0.44 (80% power, two-sided *α* = 0.05, *n* = 38), and this value refers to a single unadjusted correlation. Sex, BMI, and physical activity were not controlled in the bivariate analyses; non-significant baseline differences do not establish equivalence, and the 2-back condition reported more weekly physical activity (295 ± 199 vs. 226 ± 95.1 min/week). The associations with trunk coronal ROM and elevation at midswing should therefore be considered exploratory. Finally, the correlational design does not establish causality, and the sample of healthy young adults limits generalizability. The separate bivariate correlations do not determine whether perceived mental and physical fatigue explain unique variance in gait changes. Future adequately powered studies should examine their independent contributions using multivariable models. Also, we did not assess actual trips, slips, or falls; conclusions about fall risk therefore remain indirect.

The present assessment already included lateral step variability and several segment-based kinematic parameters. Future studies should complement these measures with more direct indicators of dynamic stability, including minimum toe clearance, mediolateral margin of stability, and center-of-mass displacement, as well as a more comprehensive assessment of lower-limb joint kinematics and muscle activity.

## 5 Conclusion and implications

The present study provides exploratory evidence that perceived fatigue and energy states are selectively associated with pre-to-post changes in spatio-temporal gait characteristics in healthy young adults. Most exploratory correlations between subjective states and spatio-temporal gait parameters did not reach nominal significance, indicating no widespread pattern of fatigue–gait associations. However, exploratory associations were observed for trunk coronal ROM and elevation at midswing. The associations between fatigue and energy ratings and trunk coronal ROM may reflect altered lateral trunk movement or normal movement variability and should not be interpreted as evidence of impaired stability. Similarly, we observed a small-to-moderate exploratory association between perceived mental fatigue and elevation at midswing during downhill walking. Although this pattern may reflect a cautious foot-trajectory strategy, it does not establish a safer or compensatory gait adaptation. The functional relevance of these associations remains uncertain because we did not directly measure dynamic stability, minimum toe clearance, or fall risk. Because the analyses were correlational, the findings do not establish that changes in perceived fatigue caused the observed pre-to-post changes in gait. The increase in perceived mental fatigue without a consistent deterioration in reaction time or accuracy indicates a dissociation between perceived mental fatigue and cognitive performance fatigability, with the present findings supporting the former but not the latter.

Overall, the findings suggest selective exploratory associations between subjective fatigue and energy states and changes in spatio-temporal gait parameters rather than widespread fatigue-related gait impairment. Given the small sample, large number of comparisons, and unadjusted *p*-values (no association remained significant after false-discovery-rate correction), the observed associations may reflect chance and require independent confirmation. The available evidence is therefore insufficient to determine whether the observed patterns represent functional adaptations, improve stability, or reduce the risk of tripping and falling. The sensitivity analysis showed that the sample provided 80% power only for correlations of approximately |*r*| ≥ 0.44; the midswing association (*r* = 0.329) was below this benchmark, and its wide confidence interval indicates limited precision. The findings should therefore be considered exploratory and require confirmation in a larger, adequately powered sample.

Future studies should replicate these findings using adequately powered, preregistered pre-post designs with prespecified primary outcomes and appropriate correction for multiple comparisons. Subjective fatigue ratings should be combined with objective measures of cognitive performance fatigability. The present assessment already included lateral step variability and several segment-based kinematic parameters. These measures should be complemented by more direct indicators of dynamic stability, including minimum toe clearance, mediolateral margin of stability, and center-of-mass displacement. A more comprehensive assessment of lower-limb joint kinematics and muscle activity could further clarify the biomechanical mechanisms associated with variations in trunk coronal ROM and midswing elevation. Randomized or counterbalanced comparisons of level, uphill, and downhill walking could determine whether these associations vary across walking conditions with different motor demands. Including single- and dual-task walking conditions would further clarify whether associations between perceived fatigue and gait become more pronounced when cognitive resources are concurrently required. Finally, comparisons across different age groups and fatigue states could determine whether the observed movement patterns are specific to young adults or differ with advancing age. Further research using larger samples, correction for multiple comparisons, and direct measures of dynamic stability is therefore needed before interpreting the observed associations as compensatory mechanisms or linking them to fall prevention. Larger studies should also examine the robustness of the associations using prespecified covariate-adjusted models (sex, BMI, physical activity) and multivariable models that separate perceived mental from physical fatigue.

## Data Availability

The raw data supporting the conclusions of this article will be made available by the authors, without undue reservation.
