# Supplementary material for: Exploring associations of perceived mental fatigue and perceived energy with gait characteristics during level and downhill walking in healthy young adults
Source: Front Sports Act Living. 2026 Oct 23;8:1973398. doi: 10.3389/fspor.2026.1973398 (PMC13739701; doi:10.3389/fspor.2026.1973398)
Supplement: Supplementary file 1 [file Supplementaryfile1.docx]

Suppl. Material A: Correlations between subjective fatigue ratings and gait parameters in the straight walking condition.

| **Subjective  fatigue ratings** | |  | | **Spatio-temporal  gait parameter** | | **Pearson's r** | | **p** | | **Lower 95% CI** | | **Upper 95% CI** | | **Effect size  (Fisher's z)** | |
| --- | --- | --- | --- | --- | --- | --- | --- | --- | --- | --- | --- | --- | --- | --- | --- |
| Physical_Energy |  | - |  | delta_Duration |  | -0.028 |  | 0.866 |  | -0.345 |  | 0.294 |  | -0.028 |  |
|  |  | - |  | delta_Cadence |  | -0.025 |  | 0.884 |  | -0.342 |  | 0.297 |  | -0.025 |  |
|  |  | - |  | delta_Double Support |  | -0.030 |  | 0.860 |  | -0.346 |  | 0.293 |  | -0.030 |  |
|  |  | - |  | delta_Elevation at Midswing |  | 0.016 |  | 0.922 |  | -0.305 |  | 0.334 |  | 0.016 |  |
|  |  | - |  | delta_Gait Cycle Duration |  | 0.004 |  | 0.980 |  | -0.316 |  | 0.324 |  | 0.004 |  |
|  |  | - |  | delta_Gait Speed |  | -0.168 |  | 0.313 |  | -0.463 |  | 0.160 |  | -0.170 |  |
|  |  | - |  | delta_Lateral Step Variability |  | -0.146 |  | 0.381 |  | -0.445 |  | 0.182 |  | -0.147 |  |
|  |  | - |  | delta_Circumduction |  | -0.287 |  | 0.080 |  | -0.556 |  | 0.036 |  | -0.296 |  |
|  |  | - |  | delta_N |  | -0.383* |  | 0.018 |  | -0.626 |  | -0.072 |  | -0.404 |  |
|  |  | - |  | delta_Foot Strike Angle |  | -0.135 |  | 0.418 |  | -0.436 |  | 0.193 |  | -0.136 |  |
|  |  | - |  | delta_Toe Off Angle |  | 0.105 |  | 0.530 |  | -0.222 |  | 0.411 |  | 0.105 |  |
|  |  | - |  | delta_Single Limb Support |  | 0.047 |  | 0.780 |  | -0.277 |  | 0.361 |  | 0.047 |  |
|  |  | - |  | delta_Stance |  | -0.012 |  | 0.943 |  | -0.330 |  | 0.309 |  | -0.012 |  |
|  |  | - |  | delta_Step Duration |  | 0.065 |  | 0.698 |  | -0.260 |  | 0.377 |  | 0.065 |  |
|  |  | - |  | delta_Stride Length |  | -0.055 |  | 0.744 |  | -0.368 |  | 0.270 |  | -0.055 |  |
|  |  | - |  | delta_Swing |  | 0.012 |  | 0.943 |  | -0.309 |  | 0.330 |  | 0.012 |  |
|  |  | - |  | delta_Terminal Double Support |  | -0.003 |  | 0.984 |  | -0.323 |  | 0.317 |  | -0.003 |  |
|  |  | - |  | delta_Toe Out Angle |  | 0.077 |  | 0.648 |  | -0.249 |  | 0.387 |  | 0.077 |  |
|  |  | - |  | delta_Lumbar Coronal ROM |  | 0.170 |  | 0.344 |  | -0.184 |  | 0.485 |  | 0.172 |  |
|  |  | - |  | delta_Lumbar Sagittal ROM |  | -0.044 |  | 0.807 |  | -0.382 |  | 0.304 |  | -0.044 |  |
|  |  | - |  | delta_Lumbar Transverse ROM |  | 0.316 |  | 0.073 |  | -0.031 |  | 0.595 |  | 0.327 |  |
|  |  | - |  | delta_TrunkCoronal ROM |  | -0.221 |  | 0.182 |  | -0.505 |  | 0.106 |  | -0.225 |  |
|  |  | - |  | delta_TrunkSagittal ROM |  | -0.180 |  | 0.278 |  | -0.473 |  | 0.148 |  | -0.182 |  |
|  |  | - |  | delta_TrunkTransverse ROM |  | -0.183 |  | 0.271 |  | -0.475 |  | 0.145 |  | -0.185 |  |
|  |  | - |  | delta_Arm Swing Velocity |  | -0.126 |  | 0.452 |  | -0.428 |  | 0.202 |  | -0.126 |  |
|  |  | - |  | delta_Arm ROM |  | -0.109 |  | 0.513 |  | -0.415 |  | 0.218 |  | -0.110 |  |
| Physical_Fatigue |  | - |  | delta_Duration |  | 0.056 |  | 0.740 |  | -0.269 |  | 0.369 |  | 0.056 |  |
|  |  | - |  | delta_Cadence |  | 0.137 |  | 0.411 |  | -0.191 |  | 0.438 |  | 0.138 |  |
|  |  | - |  | delta_Double Support |  | 0.101 |  | 0.546 |  | -0.226 |  | 0.408 |  | 0.101 |  |
|  |  | - |  | delta_Elevation at Midswing |  | -0.004 |  | 0.981 |  | -0.323 |  | 0.316 |  | -0.004 |  |
|  |  | - |  | delta_Gait Cycle Duration |  | -0.097 |  | 0.561 |  | -0.404 |  | 0.229 |  | -0.098 |  |
|  |  | - |  | delta_Gait Speed |  | 0.208 |  | 0.210 |  | -0.120 |  | 0.495 |  | 0.211 |  |
|  |  | - |  | delta_Lateral Step Variability |  | 0.269 |  | 0.103 |  | -0.056 |  | 0.542 |  | 0.275 |  |
|  |  | - |  | delta_Circumduction |  | 0.250 |  | 0.130 |  | -0.076 |  | 0.527 |  | 0.255 |  |
|  |  | - |  | delta_N |  | 0.193 |  | 0.246 |  | -0.135 |  | 0.483 |  | 0.195 |  |
|  |  | - |  | delta_Foot Strike Angle |  | 0.062 |  | 0.712 |  | -0.263 |  | 0.374 |  | 0.062 |  |
|  |  | - |  | delta_Toe Off Angle |  | 0.030 |  | 0.859 |  | -0.293 |  | 0.346 |  | 0.030 |  |
|  |  | - |  | delta_Single Limb Support |  | -0.123 |  | 0.462 |  | -0.426 |  | 0.205 |  | -0.124 |  |
|  |  | - |  | delta_Stance |  | 0.078 |  | 0.643 |  | -0.248 |  | 0.388 |  | 0.078 |  |
|  |  | - |  | delta_Step Duration |  | -0.152 |  | 0.363 |  | -0.450 |  | 0.176 |  | -0.153 |  |
|  |  | - |  | delta_Stride Length |  | 0.020 |  | 0.907 |  | -0.302 |  | 0.337 |  | 0.020 |  |
|  |  | - |  | delta_Swing |  | -0.077 |  | 0.644 |  | -0.388 |  | 0.248 |  | -0.078 |  |
|  |  | - |  | delta_Terminal Double Support |  | 0.079 |  | 0.638 |  | -0.247 |  | 0.389 |  | 0.079 |  |
|  |  | - |  | delta_Toe Out Angle |  | -0.070 |  | 0.678 |  | -0.381 |  | 0.256 |  | -0.070 |  |
|  |  | - |  | delta_Lumbar Coronal ROM |  | -0.209 |  | 0.243 |  | -0.516 |  | 0.144 |  | -0.212 |  |
|  |  | - |  | delta_Lumbar Sagittal ROM |  | -0.012 |  | 0.947 |  | -0.354 |  | 0.333 |  | -0.012 |  |
|  |  | - |  | delta_Lumbar Transverse ROM |  | -0.300 |  | 0.090 |  | -0.583 |  | 0.048 |  | -0.310 |  |
|  |  | - |  | delta_TrunkCoronal ROM |  | 0.128 |  | 0.445 |  | -0.200 |  | 0.430 |  | 0.128 |  |
|  |  | - |  | delta_TrunkSagittal ROM |  | 0.014 |  | 0.933 |  | -0.307 |  | 0.332 |  | 0.014 |  |
|  |  | - |  | delta_TrunkTransverse ROM |  | 0.012 |  | 0.941 |  | -0.308 |  | 0.331 |  | 0.012 |  |
|  |  | - |  | delta_Arm Swing Velocity |  | 0.192 |  | 0.248 |  | -0.136 |  | 0.482 |  | 0.195 |  |
|  |  | - |  | delta_Arm ROM |  | 0.112 |  | 0.502 |  | -0.215 |  | 0.417 |  | 0.113 |  |
|  |  |  |  |  |  |  |  |  |  |  |  |  |  |  |  |
| Mental_Energy |  | - |  | delta_Duration |  | -0.166 |  | 0.319 |  | -0.461 |  | 0.162 |  | -0.168 |  |
|  |  | - |  | delta_Cadence |  | 0.085 |  | 0.613 |  | -0.241 |  | 0.394 |  | 0.085 |  |
|  |  | - |  | delta_Double Support |  | -0.022 |  | 0.894 |  | -0.340 |  | 0.299 |  | -0.022 |  |
|  |  | - |  | delta_Elevation at Midswing |  | <0.001 |  | 0.995 |  | -0.319 |  | 0.321 |  | <0.001 |  |
|  |  | - |  | delta_Gait Cycle Duration |  | -0.082 |  | 0.623 |  | -0.392 |  | 0.244 |  | -0.082 |  |
|  |  | - |  | delta_Gait Speed |  | -0.032 |  | 0.849 |  | -0.348 |  | 0.291 |  | -0.032 |  |
|  |  | - |  | delta_Lateral Step Variability |  | -0.049 |  | 0.772 |  | -0.363 |  | 0.275 |  | -0.049 |  |
|  |  | - |  | delta_Circumduction |  | -0.195 |  | 0.240 |  | -0.485 |  | 0.133 |  | -0.198 |  |
|  |  | - |  | delta_N |  | -0.377* |  | 0.020 |  | -0.621 |  | -0.065 |  | -0.396 |  |
|  |  | - |  | delta_Foot Strike Angle |  | -0.114 |  | 0.496 |  | -0.418 |  | 0.214 |  | -0.114 |  |
|  |  | - |  | delta_Toe Off Angle |  | 0.088 |  | 0.599 |  | -0.238 |  | 0.397 |  | 0.088 |  |
|  |  | - |  | delta_Single Limb Support |  | 0.025 |  | 0.880 |  | -0.297 |  | 0.342 |  | 0.025 |  |
|  |  | - |  | delta_Stance |  | -0.019 |  | 0.908 |  | -0.337 |  | 0.302 |  | -0.019 |  |
|  |  | - |  | delta_Step Duration |  | -0.087 |  | 0.606 |  | -0.395 |  | 0.240 |  | -0.087 |  |
|  |  | - |  | delta_Stride Length |  | -0.068 |  | 0.683 |  | -0.380 |  | 0.257 |  | -0.068 |  |
|  |  | - |  | delta_Swing |  | 0.020 |  | 0.907 |  | -0.302 |  | 0.337 |  | 0.020 |  |
|  |  | - |  | delta_Terminal Double Support |  | 0.008 |  | 0.962 |  | -0.313 |  | 0.327 |  | 0.008 |  |
|  |  | - |  | delta_Toe Out Angle |  | 0.124 |  | 0.459 |  | -0.204 |  | 0.427 |  | 0.125 |  |
|  |  | - |  | delta_Lumbar Coronal ROM |  | 0.109 |  | 0.545 |  | -0.243 |  | 0.436 |  | 0.110 |  |
|  |  | - |  | delta_Lumbar Sagittal ROM |  | 0.085 |  | 0.637 |  | -0.266 |  | 0.416 |  | 0.085 |  |
|  |  | - |  | delta_Lumbar Transverse ROM |  | 0.225 |  | 0.208 |  | -0.128 |  | 0.527 |  | 0.229 |  |
|  |  | - |  | delta_TrunkCoronal ROM |  | -0.345* |  | 0.034 |  | -0.599 |  | -0.029 |  | -0.360 |  |
|  |  | - |  | delta_TrunkSagittal ROM |  | -0.209 |  | 0.208 |  | -0.496 |  | 0.119 |  | -0.212 |  |
|  |  | - |  | delta_TrunkTransverse ROM |  | -0.280 |  | 0.088 |  | -0.551 |  | 0.043 |  | -0.288 |  |
|  |  | - |  | delta_Arm Swing Velocity |  | -0.022 |  | 0.896 |  | -0.339 |  | 0.300 |  | -0.022 |  |
|  |  | - |  | delta_Arm ROM |  | 0.040 |  | 0.813 |  | -0.283 |  | 0.355 |  | 0.040 |  |
|  |  |  |  |  |  |  |  |  |  |  |  |  |  |  |  |
| Mental_Fatigue |  | - |  | delta_Duration |  | 0.242 |  | 0.144 |  | -0.084 |  | 0.521 |  | 0.247 |  |
|  |  | - |  | delta_Cadence |  | -0.032 |  | 0.847 |  | -0.348 |  | 0.290 |  | -0.032 |  |
|  |  | - |  | delta_Double Support |  | 0.103 |  | 0.538 |  | -0.224 |  | 0.409 |  | 0.103 |  |
|  |  | - |  | delta_Elevation at Midswing |  | -0.019 |  | 0.910 |  | -0.337 |  | 0.303 |  | -0.019 |  |
|  |  | - |  | delta_Gait Cycle Duration |  | 0.052 |  | 0.754 |  | -0.272 |  | 0.366 |  | 0.053 |  |
|  |  | - |  | delta_Gait Speed |  | 0.117 |  | 0.486 |  | -0.211 |  | 0.421 |  | 0.117 |  |
|  |  | - |  | delta_Lateral Step Variability |  | 0.081 |  | 0.628 |  | -0.245 |  | 0.391 |  | 0.081 |  |
|  |  | - |  | delta_Circumduction |  | 0.249 |  | 0.131 |  | -0.076 |  | 0.527 |  | 0.255 |  |
|  |  | - |  | delta_N |  | 0.229 |  | 0.166 |  | -0.097 |  | 0.512 |  | 0.234 |  |
|  |  | - |  | delta_Foot Strike Angle |  | 0.142 |  | 0.395 |  | -0.186 |  | 0.442 |  | 0.143 |  |
|  |  | - |  | delta_Toe Off Angle |  | -0.106 |  | 0.527 |  | -0.412 |  | 0.221 |  | -0.106 |  |
|  |  | - |  | delta_Single Limb Support |  | -0.113 |  | 0.501 |  | -0.417 |  | 0.215 |  | -0.113 |  |
|  |  | - |  | delta_Stance |  | 0.092 |  | 0.582 |  | -0.234 |  | 0.400 |  | 0.092 |  |
|  |  | - |  | delta_Step Duration |  | 0.058 |  | 0.731 |  | -0.267 |  | 0.370 |  | 0.058 |  |
|  |  | - |  | delta_Stride Length |  | 0.090 |  | 0.591 |  | -0.236 |  | 0.398 |  | 0.090 |  |
|  |  | - |  | delta_Swing |  | -0.092 |  | 0.582 |  | -0.400 |  | 0.234 |  | -0.092 |  |
|  |  | - |  | delta_Terminal Double Support |  | 0.082 |  | 0.623 |  | -0.244 |  | 0.392 |  | 0.083 |  |
|  |  | - |  | delta_Toe Out Angle |  | -0.064 |  | 0.702 |  | -0.376 |  | 0.261 |  | -0.064 |  |
|  |  | - |  | delta_Lumbar Coronal ROM |  | -0.106 |  | 0.556 |  | -0.434 |  | 0.246 |  | -0.107 |  |
|  |  | - |  | delta_Lumbar Sagittal ROM |  | -0.126 |  | 0.484 |  | -0.450 |  | 0.227 |  | -0.127 |  |
|  |  | - |  | delta_Lumbar Transverse ROM |  | -0.300 |  | 0.090 |  | -0.583 |  | 0.048 |  | -0.310 |  |
|  |  | - |  | delta_TrunkCoronal ROM |  | 0.287 |  | 0.081 |  | -0.036 |  | 0.556 |  | 0.295 |  |
|  |  | - |  | delta_TrunkSagittal ROM |  | 0.186 |  | 0.263 |  | -0.142 |  | 0.478 |  | 0.188 |  |
|  |  | - |  | delta_TrunkTransverse ROM |  | 0.315 |  | 0.054 |  | -0.005 |  | 0.577 |  | 0.326 |  |
|  |  | - |  | delta_Arm Swing Velocity |  | -0.038 |  | 0.821 |  | -0.353 |  | 0.285 |  | -0.038 |  |
|  |  | - |  | delta_Arm ROM |  | -0.175 |  | 0.292 |  | -0.469 |  | 0.153 |  | -0.177 |  |

Note: * p < .05; ** p < .01; *** p < .001; ROM = Range of Motion; delta = Change between pre and post test

Suppl. Material B: Correlations between subjective fatigue ratings and gait parameters in the 9 degree downhill walking condition.

| **Subjective  fatigue ratings** | |  | | **Spatio-temporal  gait parameter** | | **Pearson's r** | | **p** | | **Lower 95% CI** | | **Upper 95% CI** | | **Effect size  (Fisher's z)** | |
| --- | --- | --- | --- | --- | --- | --- | --- | --- | --- | --- | --- | --- | --- | --- | --- |
| Physical_Energy |  | - |  | delta_Duration |  | -0.218 |  | 0.189 |  | -0.503 |  | 0.109 |  | -0.221 |  |
|  |  | - |  | delta_Cadence |  | -0.057 |  | 0.732 |  | -0.370 |  | 0.267 |  | -0.057 |  |
|  |  | - |  | delta_Double Support |  | -0.067 |  | 0.690 |  | -0.378 |  | 0.258 |  | -0.067 |  |
|  |  | - |  | delta_Elevation at Midswing |  | -0.158 |  | 0.342 |  | -0.455 |  | 0.170 |  | -0.160 |  |
|  |  | - |  | delta_Gait Cycle Duration |  | 0.036 |  | 0.829 |  | -0.287 |  | 0.352 |  | 0.036 |  |
|  |  | - |  | delta_Gait Speed |  | -0.214 |  | 0.197 |  | -0.499 |  | 0.114 |  | -0.217 |  |
|  |  | - |  | delta_Lateral Step Variability |  | 0.213 |  | 0.199 |  | -0.115 |  | 0.499 |  | 0.216 |  |
|  |  | - |  | delta_Circumduction |  | -0.291 |  | 0.076 |  | -0.559 |  | 0.031 |  | -0.300 |  |
|  |  | - |  | delta_N |  | -0.359* |  | 0.027 |  | -0.609 |  | -0.044 |  | -0.375 |  |
|  |  | - |  | delta_Foot Strike Angle |  | 0.036 |  | 0.828 |  | -0.287 |  | 0.352 |  | 0.036 |  |
|  |  | - |  | delta_Toe Off Angle |  | 0.174 |  | 0.297 |  | -0.155 |  | 0.467 |  | 0.175 |  |
|  |  | - |  | delta_Single Limb Support |  | 0.083 |  | 0.619 |  | -0.243 |  | 0.392 |  | 0.083 |  |
|  |  | - |  | delta_Stance |  | -0.051 |  | 0.762 |  | -0.365 |  | 0.273 |  | -0.051 |  |
|  |  | - |  | delta_Step Duration |  | -0.022 |  | 0.897 |  | -0.339 |  | 0.300 |  | -0.022 |  |
|  |  | - |  | delta_Stride Length |  | -0.077 |  | 0.647 |  | -0.387 |  | 0.249 |  | -0.077 |  |
|  |  | - |  | delta_Swing |  | 0.052 |  | 0.758 |  | -0.272 |  | 0.365 |  | 0.052 |  |
|  |  | - |  | delta_Terminal Double Support |  | -0.077 |  | 0.644 |  | -0.387 |  | 0.248 |  | -0.078 |  |
|  |  | - |  | delta_Toe Out Angle |  | 0.146 |  | 0.381 |  | -0.182 |  | 0.445 |  | 0.147 |  |
|  |  | - |  | delta_Lumbar Coronal ROM |  | -0.220 |  | 0.219 |  | -0.524 |  | 0.133 |  | -0.224 |  |
|  |  | - |  | delta_Lumbar Sagittal ROM |  | 0.114 |  | 0.529 |  | -0.239 |  | 0.440 |  | 0.114 |  |
|  |  | - |  | delta_Lumbar Transverse ROM |  | 0.076 |  | 0.674 |  | -0.274 |  | 0.409 |  | 0.076 |  |
|  |  | - |  | delta_TrunkCoronal ROM |  | -0.452** |  | 0.004 |  | -0.674 |  | -0.155 |  | -0.487 |  |
|  |  | - |  | delta_TrunkSagittal ROM |  | -0.015 |  | 0.928 |  | -0.333 |  | 0.306 |  | -0.015 |  |
|  |  | - |  | delta_TrunkTransverse ROM |  | 0.129 |  | 0.442 |  | -0.199 |  | 0.431 |  | 0.129 |  |
|  |  | - |  | delta_Arm Swing Velocity |  | -0.225 |  | 0.174 |  | -0.508 |  | 0.102 |  | -0.229 |  |
|  |  | - |  | delta_Arm ROM |  | -0.213 |  | 0.199 |  | -0.499 |  | 0.115 |  | -0.216 |  |
| Physical_Fatigue |  | - |  | delta_Duration |  | 0.109 |  | 0.516 |  | -0.219 |  | 0.414 |  | 0.109 |  |
|  |  | - |  | delta_Cadence |  | 0.048 |  | 0.777 |  | -0.276 |  | 0.362 |  | 0.048 |  |
|  |  | - |  | delta_Double Support |  | 0.065 |  | 0.698 |  | -0.260 |  | 0.377 |  | 0.065 |  |
|  |  | - |  | delta_Elevation at Midswing |  | 0.187 |  | 0.262 |  | -0.141 |  | 0.478 |  | 0.189 |  |
|  |  | - |  | delta_Gait Cycle Duration |  | 0.009 |  | 0.956 |  | -0.311 |  | 0.328 |  | 0.009 |  |
|  |  | - |  | delta_Gait Speed |  | 0.216 |  | 0.193 |  | -0.112 |  | 0.501 |  | 0.219 |  |
|  |  | - |  | delta_Lateral Step Variability |  | -0.155 |  | 0.354 |  | -0.452 |  | 0.174 |  | -0.156 |  |
|  |  | - |  | delta_Circumduction |  | 0.218 |  | 0.188 |  | -0.109 |  | 0.503 |  | 0.222 |  |
|  |  | - |  | delta_N |  | 0.236 |  | 0.154 |  | -0.090 |  | 0.517 |  | 0.241 |  |
|  |  | - |  | delta_Foot Strike Angle |  | -0.158 |  | 0.344 |  | -0.454 |  | 0.171 |  | -0.159 |  |
|  |  | - |  | delta_Toe Off Angle |  | -0.007 |  | 0.969 |  | -0.326 |  | 0.314 |  | -0.007 |  |
|  |  | - |  | delta_Single Limb Support |  | -0.084 |  | 0.614 |  | -0.394 |  | 0.242 |  | -0.085 |  |
|  |  | - |  | delta_Stance |  | 0.047 |  | 0.779 |  | -0.277 |  | 0.361 |  | 0.047 |  |
|  |  | - |  | delta_Step Duration |  | -0.014 |  | 0.935 |  | -0.332 |  | 0.307 |  | -0.014 |  |
|  |  | - |  | delta_Stride Length |  | 0.086 |  | 0.609 |  | -0.241 |  | 0.395 |  | 0.086 |  |
|  |  | - |  | delta_Swing |  | -0.047 |  | 0.778 |  | -0.362 |  | 0.276 |  | -0.047 |  |
|  |  | - |  | delta_Terminal Double Support |  | 0.067 |  | 0.689 |  | -0.258 |  | 0.379 |  | 0.067 |  |
|  |  | - |  | delta_Toe Out Angle |  | -0.112 |  | 0.502 |  | -0.417 |  | 0.215 |  | -0.113 |  |
|  |  | - |  | delta_Lumbar Coronal ROM |  | 0.161 |  | 0.371 |  | -0.193 |  | 0.478 |  | 0.162 |  |
|  |  | - |  | delta_Lumbar Sagittal ROM |  | -0.145 |  | 0.420 |  | -0.465 |  | 0.209 |  | -0.146 |  |
|  |  | - |  | delta_Lumbar Transverse ROM |  | -0.221 |  | 0.217 |  | -0.524 |  | 0.133 |  | -0.224 |  |
|  |  | - |  | delta_TrunkCoronal ROM |  | 0.344* |  | 0.035 |  | 0.027 |  | 0.598 |  | 0.358 |  |
|  |  | - |  | delta_TrunkSagittal ROM |  | 0.033 |  | 0.843 |  | -0.289 |  | 0.349 |  | 0.033 |  |
|  |  | - |  | delta_TrunkTransverse ROM |  | -0.148 |  | 0.376 |  | -0.446 |  | 0.181 |  | -0.149 |  |
|  |  | - |  | delta_Arm Swing Velocity |  | 0.163 |  | 0.329 |  | -0.165 |  | 0.459 |  | 0.164 |  |
|  |  | - |  | delta_Arm ROM |  | 0.128 |  | 0.444 |  | -0.200 |  | 0.430 |  | 0.129 |  |
|  |  |  |  |  |  |  |  |  |  |  |  |  |  |  |  |
| Mental_Energy |  | - |  | delta_Duration |  | -0.054 |  | 0.745 |  | -0.368 |  | 0.270 |  | -0.054 |  |
|  |  | - |  | delta_Cadence |  | -0.082 |  | 0.623 |  | -0.392 |  | 0.244 |  | -0.082 |  |
|  |  | - |  | delta_Double Support |  | 0.162 |  | 0.331 |  | -0.166 |  | 0.458 |  | 0.164 |  |
|  |  | - |  | delta_Elevation at Midswing |  | -0.252 |  | 0.127 |  | -0.529 |  | 0.073 |  | -0.258 |  |
|  |  | - |  | delta_Gait Cycle Duration |  | 0.107 |  | 0.521 |  | -0.220 |  | 0.413 |  | 0.108 |  |
|  |  | - |  | delta_Gait Speed |  | -0.122 |  | 0.464 |  | -0.425 |  | 0.205 |  | -0.123 |  |
|  |  | - |  | delta_Lateral Step Variability |  | 0.047 |  | 0.781 |  | -0.277 |  | 0.361 |  | 0.047 |  |
|  |  | - |  | delta_Circumduction |  | -0.170 |  | 0.309 |  | -0.464 |  | 0.159 |  | -0.171 |  |
|  |  | - |  | delta_N |  | -0.329* |  | 0.043 |  | -0.587 |  | -0.011 |  | -0.342 |  |
|  |  | - |  | delta_Foot Strike Angle |  | 0.041 |  | 0.805 |  | -0.282 |  | 0.356 |  | 0.041 |  |
|  |  | - |  | delta_Toe Off Angle |  | 0.133 |  | 0.425 |  | -0.195 |  | 0.435 |  | 0.134 |  |
|  |  | - |  | delta_Single Limb Support |  | -0.152 |  | 0.362 |  | -0.450 |  | 0.176 |  | -0.153 |  |
|  |  | - |  | delta_Stance |  | 0.166 |  | 0.319 |  | -0.162 |  | 0.461 |  | 0.167 |  |
|  |  | - |  | delta_Step Duration |  | 0.016 |  | 0.922 |  | -0.305 |  | 0.334 |  | 0.016 |  |
|  |  | - |  | delta_Stride Length |  | -0.017 |  | 0.918 |  | -0.335 |  | 0.304 |  | -0.017 |  |
|  |  | - |  | delta_Swing |  | -0.165 |  | 0.322 |  | -0.460 |  | 0.163 |  | -0.167 |  |
|  |  | - |  | delta_Terminal Double Support |  | 0.141 |  | 0.397 |  | -0.187 |  | 0.441 |  | 0.142 |  |
|  |  | - |  | delta_Toe Out Angle |  | 0.142 |  | 0.396 |  | -0.186 |  | 0.441 |  | 0.143 |  |
|  |  | - |  | delta_Lumbar Coronal ROM |  | -0.149 |  | 0.406 |  | -0.469 |  | 0.204 |  | -0.151 |  |
|  |  | - |  | delta_Lumbar Sagittal ROM |  | 0.225 |  | 0.209 |  | -0.128 |  | 0.527 |  | 0.229 |  |
|  |  | - |  | delta_Lumbar Transverse ROM |  | 0.111 |  | 0.538 |  | -0.241 |  | 0.438 |  | 0.112 |  |
|  |  | - |  | delta_TrunkCoronal ROM |  | -0.401* |  | 0.013 |  | -0.639 |  | -0.093 |  | -0.425 |  |
|  |  | - |  | delta_TrunkSagittal ROM |  | -0.023 |  | 0.892 |  | -0.340 |  | 0.299 |  | -0.023 |  |
|  |  | - |  | delta_TrunkTransverse ROM |  | 0.077 |  | 0.648 |  | -0.249 |  | 0.387 |  | 0.077 |  |
|  |  | - |  | delta_Arm Swing Velocity |  | -0.238 |  | 0.151 |  | -0.518 |  | 0.089 |  | -0.242 |  |
|  |  | - |  | delta_Arm ROM |  | -0.172 |  | 0.301 |  | -0.466 |  | 0.156 |  | -0.174 |  |
|  |  |  |  |  |  |  |  |  |  |  |  |  |  |  |  |
| Mental_Fatigue |  | - |  | delta_Duration |  | -0.017 |  | 0.921 |  | -0.335 |  | 0.305 |  | -0.017 |  |
|  |  | - |  | delta_Cadence |  | 0.088 |  | 0.598 |  | -0.238 |  | 0.397 |  | 0.089 |  |
|  |  | - |  | delta_Double Support |  | -0.090 |  | 0.590 |  | -0.398 |  | 0.236 |  | -0.091 |  |
|  |  | - |  | delta_Elevation at Midswing |  | 0.329* |  | 0.044 |  | 0.010 |  | 0.587 |  | 0.342 |  |
|  |  | - |  | delta_Gait Cycle Duration |  | -0.124 |  | 0.457 |  | -0.427 |  | 0.204 |  | -0.125 |  |
|  |  | - |  | delta_Gait Speed |  | 0.202 |  | 0.223 |  | -0.126 |  | 0.490 |  | 0.205 |  |
|  |  | - |  | delta_Lateral Step Variability |  | -0.024 |  | 0.887 |  | -0.341 |  | 0.298 |  | -0.024 |  |
|  |  | - |  | delta_Circumduction |  | 0.245 |  | 0.137 |  | -0.081 |  | 0.524 |  | 0.251 |  |
|  |  | - |  | delta_N |  | 0.186 |  | 0.263 |  | -0.142 |  | 0.477 |  | 0.188 |  |
|  |  | - |  | delta_Foot Strike Angle |  | -0.041 |  | 0.805 |  | -0.356 |  | 0.282 |  | -0.041 |  |
|  |  | - |  | delta_Toe Off Angle |  | -0.074 |  | 0.661 |  | -0.384 |  | 0.252 |  | -0.074 |  |
|  |  | - |  | delta_Single Limb Support |  | 0.082 |  | 0.623 |  | -0.244 |  | 0.392 |  | 0.082 |  |
|  |  | - |  | delta_Stance |  | -0.094 |  | 0.575 |  | -0.402 |  | 0.233 |  | -0.094 |  |
|  |  | - |  | delta_Step Duration |  | -0.048 |  | 0.773 |  | -0.363 |  | 0.275 |  | -0.048 |  |
|  |  | - |  | delta_Stride Length |  | 0.053 |  | 0.752 |  | -0.271 |  | 0.367 |  | 0.053 |  |
|  |  | - |  | delta_Swing |  | 0.093 |  | 0.577 |  | -0.233 |  | 0.401 |  | 0.094 |  |
|  |  | - |  | delta_Terminal Double Support |  | -0.074 |  | 0.660 |  | -0.384 |  | 0.252 |  | -0.074 |  |
|  |  | - |  | delta_Toe Out Angle |  | -0.060 |  | 0.722 |  | -0.372 |  | 0.265 |  | -0.060 |  |
|  |  | - |  | delta_Lumbar Coronal ROM |  | 0.137 |  | 0.446 |  | -0.216 |  | 0.459 |  | 0.138 |  |
|  |  | - |  | delta_Lumbar Sagittal ROM |  | -0.192 |  | 0.285 |  | -0.502 |  | 0.162 |  | -0.194 |  |
|  |  | - |  | delta_Lumbar Transverse ROM |  | -0.214 |  | 0.231 |  | -0.519 |  | 0.139 |  | -0.218 |  |
|  |  | - |  | delta_TrunkCoronal ROM |  | 0.319 |  | 0.049* |  | -0.001 |  | 0.579 |  | 0.330 |  |
|  |  | - |  | delta_TrunkSagittal ROM |  | -0.027 |  | 0.874 |  | -0.343 |  | 0.296 |  | -0.027 |  |
|  |  | - |  | delta_TrunkTransverse ROM |  | -0.111 |  | 0.507 |  | -0.416 |  | 0.216 |  | -0.111 |  |
|  |  | - |  | delta_Arm Swing Velocity |  | 0.189 |  | 0.256 |  | -0.139 |  | 0.480 |  | 0.191 |  |
|  |  | - |  | delta_Arm ROM |  | 0.095 |  | 0.572 |  | -0.232 |  | 0.402 |  | 0.095 |  |

Note: * p < .05; ** p < .01; *** p < .001; ROM = Range of Motion; delta = Change between pre and post test
