# Supplementary material for: Exploring associations of perceived mental fatigue and perceived energy with gait characteristics during level and downhill walking in healthy young adults
Source: Front Sports Act Living. 2026 Oct 23;8:1973398. doi: 10.3389/fspor.2026.1973398 (PMC13739701; doi:10.3389/fspor.2026.1973398)
Supplement: Supplementary file 2 [file Supplementaryfile2.docx]

Suppl. Material C: Operational Definitions and Units of Mobility Lab Gait Measures

| **Measures** | **Definition** |
| --- | --- |
| Gait - Lower Limb - Cadence (steps/min) | The number of steps per minute, counting steps made by both feet |
| Gait - Lower Limb - Double Support (%GCT) | The percentage of the gait cycle in which both feet are on the ground |
| Gait - Lower Limb - Elevation at Midswing L (cm) | The height of the foot sensor measured at midswing, relative to its start position while standing. |
| Gait - Lower Limb - Elevation at Midswing R (cm) | The height of the foot sensor measured at midswing, relative to its start position while standing. |
| Gait - Lower Limb - Gait Cycle Duration (s) | The duration of a full gait cycle, measured from left foot’s initial contact to the next initial contact of the left foot |
| Gait - Lower Limb - Gait Speed (m/s) | The forward speed of the subject, measured as the forward distance traveled during the gait cycle divided by the gait cycle duration |
| Gait - Lower Limb - Lateral Step Variability L (cm) | In a series of 3 consecutive foot placements of the same foot, the variability of perpendicular deviations of the middle foot placement from the line connecting the first and third |
| Gait - Lower Limb - Lateral Step Variability R (cm) | In a series of 3 consecutive foot placements of the same foot, the variability of perpendicular deviations of the middle foot placement from the line connecting the first and third |
| Gait - Lower Limb - Circumduction L (cm) | The amount that the foot travels perpendicular to forward movement while swinging forward during an individual stride |
| Gait - Lower Limb - Circumduction R (cm) | The amount that the foot travels perpendicular to forward movement while swinging forward during an individual stride |
| Gait - Lower Limb - N (#) | The number of valid gait cycles detected within the recording. |
| Gait - Lower Limb - Foot Strike Angle L (degrees) | The angle of the foot at the point of initial contact. The pitch of the foot when flat is zero and positive when the heel contacts first. |
| Gait - Lower Limb - Foot Strike Angle R (degrees) | The angle of the foot at the point of initial contact. The pitch of the foot when flat is zero and positive when the heel contacts first. |
| Gait - Lower Limb - Toe Off Angle L (degrees) | The angle of the foot as it leaves the floor at push-off. The pitch of the foot when flat is zero. |
| Gait - Lower Limb - Toe Off Angle R (degrees) | The angle of the foot as it leaves the floor at push-off. The pitch of the foot when flat is zero. |
| Gait - Lower Limb - Single Limb Support L (%GCT) | The percentage of the gait cycle in which only one foot (left) is on the ground |
| Gait - Lower Limb - Single Limb Support R (%GCT) | The percentage of the gait cycle in which only one foot (right) is on the ground |
| Gait - Lower Limb - Stance L (%GCT) | The percentage of the gait cycle in which the foot is on the ground |
| Gait - Lower Limb - Stance R (%GCT) | The percentage of the gait cycle in which the foot is on the ground |
| Gait - Lower Limb - Step Duration L (s) | The duration of a step, measured as the period from initial contact of one foot to the next initial contact of the opposite foot |
| Gait - Lower Limb - Step Duration R (s) | The duration of a step, measured as the period from initial contact of one foot to the next initial contact of the opposite foot |
| Gait - Lower Limb - Stride Length L (m) | The forward distance travelled by a foot during a gait cycle |
| Gait - Lower Limb - Stride Length R (m) | The forward distance travelled by a foot during a gait cycle |
| Gait - Lower Limb - Swing L (%GCT) | The percentage of the gait cycle in which the foot is not on the ground |
| Gait - Lower Limb - Swing R (%GCT) | The percentage of the gait cycle in which the foot is not on the ground |
| Gait - Lower Limb - Terminal Double Support L (%GCT) | The percentage of the gait cycle in which both feet are on the ground. The handedness refers to the foot that is in the forward position. |
| Gait - Lower Limb - Terminal Double Support R (%GCT) | The percentage of the gait cycle in which both feet are on the ground. The handedness refers to the foot that is in the forward position. |
| Gait - Lower Limb - Toe Out Angle L (degrees) | The lateral angle of the foot during the stance phase, relative to the forward motion of the foot during the swing phase. Positive angles indicate an outward rotation of the toes. |
| Gait - Lower Limb - Toe Out Angle R (degrees) | The lateral angle of the foot during the stance phase, relative to the forward motion of the foot during the swing phase. Positive angles indicate an outward rotation of the toes. |
| Gait - Lumbar - Coronal Range of Motion (degrees) | The angular range of the lumbar spine in the coronal plane. |
| Gait - Lumbar - Sagittal Range of Motion (degrees) | The angular range of the lumbar spine in the sagittal plane. |
| Gait - Lumbar - Transverse Range of Motion (degrees) | The angular range of the lumbar spine in the transverse plane. |
| Gait - Trunk - Coronal Range of Motion (degrees) | The angular range of trunk movement in the coronal plane during walking. |
| Gait - Trunk - Sagittal Range of Motion (degrees) | The angular range of trunk movement in the sagittal plane during walking. |
| Gait - Trunk - Transverse Range of Motion (degrees) | The angular range of trunk movement in the transverse plane during walkin |
| Gait - Arm - Swing Velocity (degrees/s) | The angular velocity of arm movement during walking. |
| Gait - Arm - Range of Motion (degrees) | The angular range of arm movement during walking. |
|  |  |
